# Comparison of a Multiple Daily Insulin Injection Regimen (Glargine or Detemir Once Daily Plus Prandial Insulin Aspart) and Continuous Subcutaneous Insulin Infusion (Aspart) in Short-Term Intensive Insulin Therapy for Poorly Controlled Type 2 Diabetes Patients

**DOI:** 10.1155/2013/614242

**Published:** 2013-05-08

**Authors:** Wen-shan Lv, Li Li, Jun-ping Wen, Rong-fang Pan, Rui-xia Sun, Jing Wang, Yu-xin Xian, Cai-xia Cao, Yan-yan Gao

**Affiliations:** ^1^The Department of Internal Medicine, The Affiliated Hospital of Medical College, Qingdao University, Qingdao 266100, China; ^2^Department of Endocrinology, Key Laboratory of Endocrinology, Fujian Provincial Hospital, Fujian Medical University, North Branch of Fujian Provincial Hospital, Fujian Provincial Geriatric Hospital, Fuzhou 350001, China

## Abstract

*Aims.* To examine the potential differences between multiple daily injection (MDI) regimens based on new long-acting insulin analogues (glargine or detemir) plus prandial insulin aspart and continuous subcutaneous insulin aspart infusion (CSII) in patients with poorly controlled type 2 diabetes. *Methods.* Patients (*n* = 119) with poorly controlled type 2 diabetes of a duration exceeding five years were randomly assigned into three groups: Group A treated with CSII using insulin aspart; Group B treated with glargine-based MDI and Group C treated with detemir-based MDI. *Results.* Good glycemic control was achieved by patients in Group A in a significantly shorter duration than patients in Groups B and C. Total daily insulin, basal insulin dose and dose per kg body weight in Group A were significantly less than those in Groups B and C. Daily blood glucose fluctuation in Group A was significantly less than that in Groups B and C. There were no differences between Groups B and C. *Conclusions.* Aspart-based CSII may achieve good blood glucose control with less insulin doses over a shorter period compared with glargine or detemir-based MDI. No differences between glargine- and detemir-based MDI were detected in poorly controlled subjects with type 2 diabetes.

## 1. Introduction

Glucotoxicity is one of the major factors involved in progressive deterioration of beta-cell function and mass in type 2 diabetes mellitus (T2DM) [1]. Beta-cell function can be improved and leaded to temporary remission in newly diagnosed T2DM treated with short-term intensive insulin therapy [1, 2]. About 68% of patients with a mean duration of 5.9 ± 6.6 years of established T2DM may achieve fasting glucose <7.0 mmol/L from antidiabetic therapy as well [3]. Short-term intensive insulin treatment even improves fibrinolytic profile and vibratory sensation in patients with T2DM [4]. Therefore, patients with long-term, poorly controlled T2DM [5], perioperative hyperglycemia [6], and diabetic patients suffering from acute coronary events [7] may all be good candidates for short-term, intensive insulin therapy.

There are three forms of intensive insulin therapy: multiple daily injection (MDI); continuous subcutaneous insulin infusion (CSII); continuous intravenous insulin infusion. In the context of intensive diabetes management, insulin pumps provide precise insulin delivery throughout the day and improve the accuracy of bolus dose calculations to closely follow the physiologic patterns of secretion observed in patients without diabetes. In this way, improved glycemic control with less frequent and severe hypoglycemic episodes can be achieved. Therefore, CSII may be the best current therapeutic option for patients with diabetes requiring multiple daily injections. However, not all patients needing insulin intensive therapy have access to insulin pump therapy due to limitations including high expense, high daily use charges, inadequate medical resources, and restrictive health insurance policies.

 Recently, the development of new insulin preparations that mimic the normal mealtime bursts of insulin such as aspart insulin and lispro insulin and the availability of new insulin preparations that simulate endogenous basal insulin more precisely such as glargine and detemir may facilitate the ability to achieve long-term control over blood glucose in patients with T2DM [8]. Currently, it is not known whether MDI regimens based on new long-acting insulin analogs such as glargine and detemir may replace the need for CSII [9, 10]. In T2DM, CSII and MDI produce similar glycemic control, but there have been a paucity of studies comparing glargine and detemir based MDI with insulin pump therapy [9]. Although it is possible that CSII may be beneficial in selected patient groups with T2DM, further study is required to elucidate this hypothesis.

 The present two-center prospective study was designed to compare the efficacy and safety of three short-term, intensive insulin therapy procedures: CSII with fasting insulin analog aspart, glargine based MDI, and detemir based MDI in patients with established T2DM of a duration exceeding five years but poorly controlled by oral antidiabetic drugs (OAD) or conventional insulin therapy. Length of time to achieve glucose goals, total daily insulin doses, per kg body weight daily insulin doses, daily basal insulin doses, per kg body weight daily basal insulin doses, daily blood glucose fluctuations, and hypoglycemic and nocturnal hypoglycemia episodes in each group were assessed in order to compare the three treatments.

## 2. Research Design and Methods

All T2DM patients (*n* = 119; 48 male, 71 female) were hospitalized between October, 2010, to October, 2011, in the Department of Internal Medicine, the Affiliated Hospital of Medical College, Qingdao University, Qingdao, China, and the Department of Endocrinology at Fujian Provincial Hospital, Fuzhou, China. Exclusion criteria included patients with acute complications, renal dysfunction (defined by serum creatinine ≥136 *μ*mol/L in males or ≥124 mol/L in females or creatinine clearance <60 mL/min), hepatic dysfunction (transaminases >2.5 × upper limit of normal serum alanine and aspartate aminotransferase levels), proliferative retinopathy, acute or chronic infection, malignancy and pregnancy. Prior to randomization, patient demographic characteristics (mean ± standard deviation (SD)) were as follows: age 61.1 ± 8.9 years, diabetes duration 10.37 ± 4.14 years, body weight 68.58 ± 10.34 kg, body mass index (BMI) 25.07 ± 2.83 kg/m^2^, glycosylated hemoglobin (HbA1c) 9.56 ± 1.58%, and initial random blood glucose 14.4 ± 3.91 mmol/L. All recruited patients participated in diabetes education programs during hospitalization and gave written informed consent before treatment. The study was approved by the Local Ethics Committee.

## 3. Study Procedures

Enrolled subjects were randomly assigned into three groups: CSII (Group A); glargine-based MDI (Group B); detemir-based MDI (Group C). During insulin intensive therapy, patients did not use any other hypoglycemic drugs. Patients in Group A were treated with aspart (Novo Nordisk, Bagsvaerd, Denmark) using Medtronic (Northridge, CA) insulin pumps. Patients in group B were treated with aspart (Novo Nordisk, Bagsvaerd, Denmark) before each meal and glargine (Lantus, Aventis Pharma, Frankfurt, Germany) at bedtime. Patients in group C were treated with aspart (Novo Nordisk, Bagsvaerd, Denmark) before each meal and detemir (Novo Nordisk, Bagsvaerd, Denmark) at bedtime. The total daily dose of insulin was calculated in international units (IU). Starting doses were determined based on a total daily dose of 0.3–0.4 IU/kg/day, with 50% provided as bolus (premeal) insulin and 50% provided as basal insulin. In Group A, initial basal dose was divided into the following six periods of the day: the period from 00:00 to 03:00 hours, 03:00 to 09:00 hours, 09:00 to 12:00 hours, 12:00 to 16:00 hours, 16:00 to 20:00 hours, and 20:00 to 24:00 hours. Premeal doses were distributed evenly into three premeals. Blood glucose was monitored by a stable blood glucose monitoring device from Roche (Accu-Chek Performa, Germany). Blood glucose was measured from finger-stick blood samples five times a day (fasting, two hours after breakfast, two hours after lunch, two hours after supper and at 3:00 a.m.). Basal insulin doses were titrated to target fasting glucose between 4.0 and 7.0 mmol/L. Premeal insulin doses were adjusted according to two hour postprandial glucose levels to achieve the target of ≤11.0 mmol/L. If the subject achieved two consecutive days at goal, length of time needed to achieve target, total daily insulin doses, daily basal insulin doses, blood glucose fluctuations, and hypoglycemia episodes were calculated. Hypoglycemic episodes were classified as *severe* hypoglycemia when patients were not able to treat the episode themselves and blood glucose was ≤3.9 mmol/L, *symptomatic* hypoglycemia when patients were able to treat the episode and blood glucose was ≤3.9 mmol/L, and *relative* hypoglycemia when patients with symptoms of hypoglycemia but blood glucose was either >3.9 mmol/L or was not measured [11]. Hypoglycemic episodes were also evaluated as all events (all episodes occurring over a 24-hour period) and nocturnal events (episodes occurring between 11 pm and 6 am).

## 4. Statistical Analyses

All statistical analyses were performed with commercial software (SPSS, version 17.0 for Windows, SPSS, Inc., Chicago, IL). Descriptive analyses of the qualitative variables were with proportions and percentages. Quantitative variables were described as mean and SD values. The comparison between groups was performed by analysis of variance. Daily blood glucose fluctuations were expressed as the ratio of the arithmetic square root of the sum of the square of differences between each time period blood glucose value and the daily mean blood glucose to daily mean blood glucose. Frequencies of hypoglycemia and nocturnal hypoglycemia were calculated by total daily hypoglycemia episodes and total nocturnal hypoglycemia episodes divided by the sum of the period of time to arrive at target in each group. A value of *P* < 0.05 was considered as statistically significant.

## 5. Results

There were no clinically relevant differences in demographic characteristics between subjects in the three treatment groups (Table 1). Patients in the CSII group reached glycemic goals in about four days (4.2 ± 1.34 days) while patients in glargine- or detemir-based MDI groups achieved glycemic goals in about seven days (7.48 ± 2.51 days and 6.85 ± 2.28 days, respectively, *P* < 0.05 for A versus B or C; *P* > 0.05 for B versus C). At the time glycemic goals were reached, total daily insulin dose and the dose per kg body weight in Group A (40.25 ± 5.90 IU, 0.60 ± 0.11 IU/kg/day) were significantly less than those in Group B (49.35 ± 8.90 IU, 0.74 ± 0.14 IU/kg/day) and Group C (49.21 ± 9.35 IU, 0.72 ± 0.17 IU/kg/day, *P* < 0.05 for A versus B or C; *P* > 0.05 for B versus C) (Table 2). Total daily basal insulin doses and the dose per kg body weight in Group A (20.09 ± 3.28 IU, 0.30 ± 0.05 IU/kg/day) were significantly less than those in Group B (22.70 ± 4.91 IU, 0.34 ± 0.07 IU/kg/day) and Group C (23.51 ± 3.99 IU, 0.34 ± 0.07 IU/kg/day, *P* < 0.05 for A versus B or C; *P* > 0.05 for B versus C), as well (Table 3).

At each time period, blood glucose levels and mean blood glucose levels were significantly lower than random blood glucose levels before intensive insulin therapy in each group (*P* < 0.05); however, at the time glycemic goals were reached, no statistical differences in fasting blood glucose levels among the three groups were detected (6.03 ± 0.47 mmol/L, 5.98 ± 0.72 mmol/L, and 6.17 ± 0.53 mmol/L, resp., *P* > 0.05) (Table 4). Additionally, no statistically significant differences in two-hour after breakfast blood glucose levels (8.77 ± 1.23 mmol/L, 8.63 ± 1.46 mmol/L, and 8.95 ± 1.22 mmol/L, resp.), two-hour after lunch blood glucose levels (9.04 ± 0.94 mmol/L, 9.52 ± 1.60 mmol/L, and 9.01 ± 1.35 mmol/L, resp.), two-hour after supper blood glucose levels (8.21 ± 0.20 mmol/L, 8.82 ± 1.52 mmol/L, and 8.57 ± 1.68 mmol/L, resp.), and 3:00 A.M. blood glucose levels (6.14 ± 0.63 mmol/L, 6.13 ± 0.87 mmol/L, and 5.85 ± 0.65 mmol/L, resp.) among the three groups (all *P* > 0.05) (Table 4) were found. Daily blood glucose fluctuations in Group A, however, (0.19 ± 0.03) were significantly less than those in Group B (0.24 ± 0.04) and Group C (0.22 ± 0.04, *P* < 0.05 for A versus B or C; *P* > 0.05 for B versus C) (Table 4).

 No severe hypoglycemic episodes were reported in any treatment group, and there were no statistically significant differences in hypoglycemic and nocturnal hypoglycemia episodes among the three groups (Group A (0.10 times/person day and 0.03 times/person day), Group B (0.07 times/person day and 0.02 times/person day), and Group C (0.05 times/person day and 0.02 times/person day), *P* > 0.05; Table 5).

## 6. Discussion

Fast-acting insulin analogues (lispro and aspart insulin) are characterized by amino acid substitutions in the C-terminal portion of *β*-chain and a fast absorption rate from the subcutaneous tissue. CSII of aspart with insulin pumps could mimic physiological insulin secretion at different periods throughout the day with faster onset and offset than subcutaneous regular insulin, allowing both prandial and corrective boluses, therefore reproducing a more physiological pattern of insulin secretion and improvements in the overall 24-hour glycemic profile. Results from the present study investigating patients with established T2DM (duration > five years) demonstrated that patients in the CSII group reached glycemic goals in a significantly shorter period of time than patients in either the glargine- or detemir-based MDI groups. Total daily insulin doses and the doses per kg body weight in Group A were significantly less than those in Groups B and C. The total daily basal insulin doses and the dose per kg body weight in Group A were significantly less than those in Groups B and C as well. Daily blood glucose fluctuations in Group A were significantly less than those in Groups B and C. These results suggest that fast-acting insulin analogue-based CSII remains the gold standard effective mode of intensive insulin therapy in T2DM.

 Unfortunately, insulin pumps are limited by high expense and complex injection protocols which increase the potential for patient errors and noncompliance. The development of fast-acting insulin analogues and long-acting insulin analogues suitable for once-daily administration may help overcome these challenges. Data generated in the present study suggest that glargine- or detemir-based MDI can reach glycemic goals in approximately seven days (glargine, 7.48 ± 2.51 days versus detemir, 6.85 ± 2.28 days, *P* > 0.05). Therefore, glargine- or detemir-based MDI may be effective and reasonable alternatives to insulin pump therapy, especially in nondiabetes specialist wards or basic hospital settings where insulin pumps are not readily available.

 The two basal insulin analogues, glargine and detemir, developed by adjusting the isoelectric point and adding a fatty acid residue, respectively, have a protracted duration of action and a relatively smooth profile. Both analogues have a longer duration of action, less of a peak of activity, and a reduced variability with repeated injections [12]. However, there is sufficient evidence to demonstrate that these two long-acting insulin analogs are different in both their pharmacokinetic and pharmacodynamic profiles [13]. Furthermore, the relative merit of the two analogs when compared to each other has been a matter of some controversy [14]. The present study found no significant differences in the time to achieve target, daily total and basal insulin doses, daily per body weight unit total and basal insulin doses between Groups B and C (*P* > 0.05) suggesting that the efficacy of detemir and glargine may be comparable in subjects with T2DM when combined with aspart in a basal-bolus regimen.

 DeVries et al. [15] reported that goal-titrated, twice-daily, basal insulin tended to increase insulin doses disproportionately with regard to improvement in glycemic control. Several studies have reported that detemir was often injected twicedaily at a higher dose than glargine (injected once-daily at a lower dose) to achieve the same level of glycemic control [16, 17]. However, other studies conducted by diabetologists reported oncedaily dosing with detemir and glargine was comparable in T2DM subjects [18, 19]. Results of most clamp studies show that duration of action with both analogues is dose dependent, and in the clinically relevant range of 0.35–0.8 U/kg, it is close to 24 hours in people with T2DM. In fact, both analogues seem to be very similar with regard to the mean shape of their pharmacodynamic profile and duration of action [14]. These findings in experimental glucose clamp studies are consistent with observations in clinical trials and support routine once-daily use with either analogue, in particular in people with T2DM [14, 20], and are further confirmed by the results of the present study.

 Glucose variability, such as intraday glucose fluctuations, contributes to oxidative stress, which has been linked to the pathogenesis of the long-term complications of diabetes [21, 22] and avoiding glucose fluctuations in diabetic patients seems to be an emerging therapeutic challenge [23]. The results of the present study demonstrate that daily blood glucose fluctuations were significantly narrower in aspart-based CSII than in glargine- or detemir-based MDI (0.19 ± 0.03, 0.24 ± 0.04, and 0.22 ± 0.04, resp.; *P* < 0.05 for A versus B or C; *P* > 0.05 for B versus C). These results are in accord with the general theory that basal insulin substitution with CSII provides less variable glucose levels than with long-acting insulin analogs in patients with diabetes [24]. The variability in blood glucose control seems particularly important with long-acting insulins. Several studies have reported that detemir shows less within-subject variability in its metabolic effects than glargine [14, 20, 25–28]. However, we found no difference in daily blood fluctuations between glargine- and detemir-based MDI in accord with the study of T2DM patients by Tone et al. [29].

 Hypoglycemia is one of the main limiting factors for patients with diabetes requiring insulin in achieving tight glycemic control and reduced rates of complications. Nocturnal hypoglycemia may be the most common type of hypoglycemia in individuals with diabetes using insulin and is particularly worrisome because it often goes undetected and may lead to unconsciousness and even death in severe cases. The results of the present study found no significant differences in hypoglycemic and nocturnal hypoglycemia episodes among the three groups (*P* > 0.05) suggesting that the safety of either glargine- or detemir-based MDI may be comparable in subjects with T2DM when compared with aspart-based CSII therapy.

 From the data generated in the present study, it would seem that fast-acting analog-based CSII could achieve good blood glucose control with fewer insulin doses over shorter periods of time compared with glargine- or detemir-based MDI which remains the most effective mode of intensive insulin therapy in poorly controlled T2DM. However, if local medical conditions and individual factors do not allow the use of insulin pumps, once daily glargine or detemir at bedtime combined with a fast-acting insulin analogue at meals should be an effective and reasonable alternative [30].

## Figures and Tables

**Table 1:** Patient demographic characteristics.

	Group A	Group B	Group C
Number	40	40	39

Gender			
Male *n* (%)	16 (40)	13 (32.5)	19 (48.7)
Female *n* (%)	24 (60)	27 (67.5)	20 (51.3)
Age (years ± SD)	61.15 ± 7.76	62.03 ± 9.33	60.1 ± 9.73
Diabetes duration (years ± SD)	10.08 ± 2.56	10.62 ± 4.32	10.41 ± 5.22
Weight (kg ± SD)	68.24 ± 9.01	67.55 ± 10.48	70.00 ± 11.52
BMI (kg/m^2^± SD)	25.00 ± 2.54	24.96 ± 3.08	25.28 ± 2.90
Waistline (cm ± SD)	92.70 ± 10.15	92.48 ± 9.91	93.22 ± 9.28
Random blood glucose (mmol/L ± SD)	14.88 ± 3.61	13.58 ± 4.12	14.88 ± 3.93
HbA1c (% ± SD)	9.86 ± 1.69	9.25 ± 1.54	9.56 ± 1.49

BMI: body mass index; HbA1c: glycosylated hemoglobin; SD: standard deviation.

**Table 2:** Comparison of periods of time to arrival at target and daily insulin doses.

Group	Period of time to arrive at target (days ± SD)	Total daily insulin doses (IU ± SD)	Per body weight unit insulin doses (IU/kg ± SD)
A	4.20 ± 1.34	40.25 ± 5.90	0.60 ± 0.11
B	7.48 ± 2.51*	49.35 ± 8.90*	0.74 ± 0.14
C	6.85 ± 2.28*	49.21 ± 9.35*	0.72 ± 0.17

*P < 0.05 versus Group A.

IU: international units; SD: standard deviation.

**Table 3:** Comparison of basal insulin doses upon achievement of good blood glucose control.

Group	Daily basal insulin doses (IU ± SD)	Per body weight unit basal insulin doses (IU/kg ± SD)
A	20.09 ± 3.28	0.30 ± 0.05
B	22.70 ± 4.91*	0.34 ± 0.07*
C	23.51 ± 3.99*	0.34 ± 0.07*

*P < 0.05 versus Group A.

IU: international units; SD: standard deviation.

**Table 4:** Comparison of blood glucose levels in mmol/L (± standard deviation) at different times and blood glucose fluctuations.

Group	Fasting	Two hours after breakfast	Two hours after lunch	Two hours after supper	3:00 A.M.	Blood glucose fluctuations
A	6.03 ± 0.47	8.77 ± 1.23	9.04 ± 0.94	8.21 ± 0.90	6.14 ± 0.63	0.19 ± 0.03
B	5.98 ± 0.72	8.63 ± 1.46	9.52 ± 1.60	8.82 ± 1.52	6.13 ± 0.87	0.24 ± 0.04*
C	6.17 ± 0.53	8.95 ± 1.22	9.01 ± 1.35	8.57 ± 1.68	5.85 ± 0.65	0.22 ± 0.04*

*P < 0.05 versus Group A.

**Table 5:** Comparison of hypoglycemia and nocturnal hypoglycemia episodes.

Group	Hypoglycemia episodes (times)	Nocturnal hypoglycemia episodes (times)	Frequency of hypoglycemia (times/person · day)	Frequency of nocturnal hypoglycemia (times/person · day)
A	17	5	0.10	0.03
B	20	7	0.07	0.02
C	13	6	0.05	0.02

## Abbreviations

BMI: Body mass index
CSII: Continuous subcutaneous insulin infusion
HbA1c: Glycosylated hemoglobin
IU: International units
MDI: Multiple daily injection
OAD: Oral antidiabetic drugs
T2DM: Type 2 diabetes mellitus.
